# Error-Tolerant Leadership in Public Health: How Tolerance From Leaders Strengthens Healthcare Workers’ Collectivism, Colleague Trust, and Job Satisfaction

**DOI:** 10.3389/ijph.2026.1608795

**Published:** 2026-02-25

**Authors:** Yuan Zhang, Jun Huang, Kui Hao

**Affiliations:** 1 Business School, Zhuhai College of Science and Technology, Zhuhai, Guangdong, China; 2 Research Department, China Innovation Finance Institute, Chengdu, Sichuan, China; 3 Outpatient Department, Zaozhuang Municipal Hospital, Zaozhuang, Shandong, China

**Keywords:** leader tolerance, job satisfaction, collectivism, trust in colleagues, COR

## Abstract

**Objectives:**

This study investigates how leader tolerance for errors improves front-line healthcare workers’ job satisfaction by actively fostering collectivism and trust in colleagues, drawing on Conservation of Resources theory. It seeks to offer practical leadership insights for public health systems operating under pressure.

**Methods:**

We conducted a cross-sectional survey of 553 healthcare professionals in a Chinese municipal hospital and analyzed the data using Hayes’ PROCESS Macro (Model 6) to test the proposed mediation model.

**Results:**

Leader tolerance significantly increases job satisfaction (*β = 0.48, p < 0.001*), with collectivism (*β = 0.18, p < 0.001*) and trust in colleagues (*β = 0.32, p < 0.001*) serving as powerful mediators. Together, these mediators account for 51.16% of the total effect, with trust in colleagues emerging as the dominant pathway mediator (30.23%).

**Conclusion:**

Tolerant leadership actively builds a psychologically safe and collaborative work climate, elevating job satisfaction and enabling clinical teams to function as adaptive learning communities. Developing leadership capabilities that normalize error tolerance and reinforce interpersonal trust is critical for strengthening public health resilience in both crisis and long-term contexts.

## Introduction

The COVID-19 pandemic exposed critical vulnerabilities in the global health workforce, exacerbating burnout and attrition among front-line professionals [1, 2]. This has intensified a key leadership dilemma: while strict error-control is necessary for patient safety, overly punitive approaches can harm staff wellbeing. Leader tolerance—an approach that treats unintentional errors as learning opportunities rather than grounds for blame [3]—has emerged as a potential catalyst for psychological safety and resilience.

However, significant research gaps persist. Existing studies often focus narrowly on nurses, neglecting multidisciplinary public health teams [4], and prioritize behavioral over psychological outcomes like job satisfaction [5, 6]. Crucially, prior research relies on simplistic direct-effect models, failing to explain the underlying psychological mechanism.

Guided by Conservation of Resources (COR) theory, this study proposes and tests a sequential mediation model: leader tolerance → collectivism → trust in colleagues → job satisfaction. This theorizes leader tolerance as initiating a resource-generating sequence, where it first builds collectivism as a social resource, which then fosters relational trust, ultimately enhancing job satisfaction. We test this chain in a multidisciplinary healthcare cohort, moving beyond corporate-based evidence [7, 8] to provide a robust, theory-driven framework.

By validating this causal pathway, our findings offer public health leaders an actionable blueprint for building resilient, psychologically safe teams capable of navigating sustained health challenges.

### Theoretical Background and Hypotheses

#### Conservation of Resource Theory

COR theory posits that individuals actively seek to acquire, retain, and protect valued resources, and experience psychological stress when these resources are threatened, depleted, or fail to yield expected returns after significant investment [9]. This framework is highly relevant to healthcare environments, where employees operate under chronic workload pressure, emotionally charged decision-making, and high accountability.

#### Leader Tolerance and Employees’ Job Satisfaction

Leader tolerance—an essential dimension of inclusive leadership—is particularly critical in public health sectors where errors carry high consequences [3, 10]. It reflects leaders’ willingness to accept and support unintentional mistakes by encouraging learning-oriented behavior before errors occur (pre-error tolerance) and offering emotional and resource support afterward (post-error tolerance) [2, 7, 8]. This dual process contributes not only to learning culture but also to patient safety and outcome quality.

From a COR perspective, tolerant leadership provides a crucial environmental resource in a domain characterized by chronic resource scarcity [11]. Persistent fear of repercussions drains healthcare professionals’ psychological and emotional resources, threatens their credibility, and encourages defensive behaviors such as error concealment [12]. In contrast, leader tolerance signals safety and organizational support [13], allowing employees to redirect finite energy away from self-preservation and toward mission-critical tasks—such as refining public health strategies or coordinating outbreak responses. This constructive redirection fosters greater emotional stability and workplace satisfaction [14]. Therefore, we hypothesize that:

H1 Leader tolerance positively predicts healthcare professionals’ job satisfaction.

#### The Mediating Role of Collectivism

We propose that leader tolerance influences job satisfaction not only directly but also indirectly by first cultivating collectivism, conceptualized here as an individual-level value orientation emphasizing shared goals, interdependence, and collective welfare [15].

Within the COR theory, collectivism represents the first stage of the resource gain spiral. When leaders tolerate mistakes, employees perceive lower interpersonal risk, which releases cognitive and emotional resources otherwise spent on self-protection. These liberated resources can then be invested in collective engagement—such as co-developing disease prevention protocols or coordinating vaccination efforts. Through such investments, shared social resources emerge, including group cohesion, reciprocity norms, and joint purpose. These collective resources enhance job satisfaction by fulfilling employees’ psychological need for belonging and buffering them against occupational stress. Thus, we hypothesize that:

H2 Collectivism mediates the positive relationship between leader tolerance and healthcare employees’ job satisfaction.

#### Mediating Role of Trust in Colleagues

We further propose trust in colleagues as a second, independent mediating mechanism. Trust is defined as confidence in colleagues’ competence and reliability (cognitive trust) and belief in their goodwill and care (affective trust) [16].

From a COR standpoint, this pathway reflects a direct relational mechanism. Leader tolerance reduces interpersonal uncertainty by modeling understanding and non-punitive behavior, signaling that risk-taking and vulnerability will not be exploited [6]. This perception enables staff to invest relational energy into trusting colleagues, especially during critical joint operations such as emergency response or large-scale public health coordination [17].

Trust, once formed, becomes a powerful relational resource—reducing verification fatigue, improving coordination efficiency, and reinforcing emotional security—thereby increasing job satisfaction [17]. We, therefore, hypothesize that:

H3 Trust in colleagues mediates the positive relationship between leader tolerance and healthcare employees’ job satisfaction.

#### Sequential Mediating Roles of Collectivism and Trust in Colleagues

We propose a sequential mediation mechanism where leader tolerance initiates a socio-relational resource gain process in healthcare teams. First, tolerant leadership provides psychological safety that enables staff to adopt collectivist values by reducing perceived risks in prioritizing collective goals during interdependent tasks. This collectivist orientation then serves as a resource investment platform, motivating cooperative behaviors, transparent knowledge sharing, and mutual assistance, which is particularly in public health scenarios. Through repeated cooperative interactions, these behaviors systematically foster trust in colleagues [18–20], which multiplies psychological resources by reducing monitoring demands and providing emotional support [21]. The accumulated resources ultimately enhance job satisfaction, leading to our hypothesis:

H4 Collectivism and trust in colleagues sequentially mediate the relationship between leader tolerance and job satisfaction.

**FIGURE 1:**
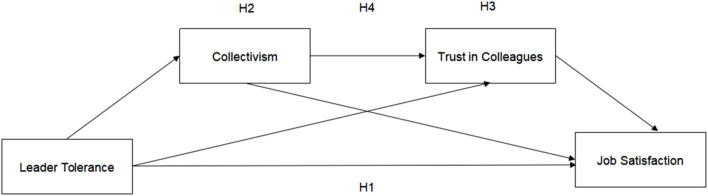
Theoretical model (Shandong, China. 2024).

Figure 1 illustrates the theoretical model.

## Methods

### Study Design and Reporting Standard

We conducted a cross-sectional study in full compliance with the Strengthening the Reporting of Observational Studies in Epidemiology (STROBE) guidelines [22]. The completed STROBE checklist for cross-sectional studies is provided as a supplementary file.

### Participants and Research Setting

This study took place at the largest tertiary public hospital in a third-tier city in Shandong Province, China. Serving approximately three million residents, it recorded one million annual outpatient visits between 2023 and 2024. This high-work load institution is representative of public hospitals in middle-income regions, where demand for services consistently exceeds available resources.

The hospital’s ongoing engagement in China’s national healthcare reform further underscores its strategic relevance as a research context [23]. These policies, while aiming to improve system efficiency but simultaneously increase operational stress and psychological demands on healthcare workers. This environment provides an ideal setting to examine whether leader tolerance can activate a resource gain cycle—first by fostering collectivism as a shared coping resource, and subsequently by strengthening trust in colleagues as a relational support mechanism. These reform pressures mirror those observed in international systems transitioning to value-based care or payment restructuring, enhancing the broader transferability of this study.

In essence, this hospital reflects the sustainability crisis currently facing the global health workforce. By investigating how leader tolerance may strengthen collectivism and trust in such a high-pressure environment, this study seeks to identify scalable leadership strategies that can reinforce workforce resilience in public health systems worldwide.

### Survey Procedure and Data Quality Assurance

We received ethical approval from the Ethics Committee of the first author’s institution, along with administrative authorization from the hospital’s Human Resource Management Department. We then distributed an online survey via the Wenjuanxing platform to all 737 eligible healthcare professionals. The cover letter clearly outlined the study’s purpose, emphasized voluntary participation, and assured complete anonymity.

We applied a rigorous, predefined multi-step data cleaning protocol to ensure analytic reliability: (1) Incomplete responses – 58 cases were excluded for missing values on key variables or demographic data, as such omissions would compromise complete case analysis; (2) Uniform responses – 112 cases were removed due to invariant responses across entire multi-item scales, indicating non-engagement or satisficing behavior; (3) Logical inconsistencies – 14 cases were excluded where reported organizational tenure exceeded the participant’s age minus 16 years, reflecting the earliest plausible employment age for vocational medical graduates in China.

We also considered the potential impact of data exclusions on sample representativeness. Although methodologically justified, the removal of uniform responses may lead to under-representation of disengaged individuals or those with extreme levels of satisfaction or dissatisfaction. As a result, the generalizability of absolute score levels to the broader healthcare workforce may be limited.

To address potential selection bias, we conducted sensitivity analyses comparing the cleaned sample against results generated from a multiply imputed dataset that retained missing and logically inconsistent cases.

### Measures

We employed validated psychometric scales to assess all study constructs. Except for the leader tolerance scale, which was originally developed in China, all instruments were translated and back-translated to ensure conceptual equivalence. Items were rated on a 5-point Likert scale (1 = strongly disagree, 5 = strongly agree).

Leader tolerance was measured using the 4-item scale developed by Zhang and Tang (2016) [3], which is contextualized for the Chinese environment. Sample items include: “When subordinates make mistakes unintentionally, my leader tolerates their errors,” and “My leader forgives errors or failures made by his or her subordinates.” The scale demonstrated high reliability (Cronbach’s α = 0.90).

Collectivism was measured using the 3-item scale designed by Doney et al. (1998) [24], such as “We consciously (indicating group orientation) value joint efforts and group rewards,” and “I interact in an interdependent, cooperative mode.” The scale showed strong reliability (Cronbach’s α = 0.87).

Trust in colleagues was assessed using Ng and Chua’s [25] 8-item scale, capturing both cognitive and affective trust. This scale encompasses two dimensions: cognitive trust and affective trust. Cognitive trust is exemplified by statements such as “I can rely on my colleagues to handle critical tasks.” Affective trust is reflected in situations where employees feel they can openly discuss challenges with colleagues. Reliability was high across subdimensions (Cronbach’s α = 0.92 cognitive; Cronbach’s α = 0.94 affective; overall Cronbach’s α = 0.95).

Job satisfaction was measured with Schriesheim and Tsui’s [26] 6-item scale, including items such as “I am satisfied with my colleagues” and “I am satisfied with my leader.” The scale exhibited solid reliability (i.e., demonstrated strong internal consistency) within recommended threshold levels (Cronbach’s α = 0.87).

We controlled for age, gender, education level, marital status, and organizational tenure, as these demographic factors may shape employees’ access to social resources and influence attitudes toward leadership and workplace relationships [27–29].

### Analytical Approach and Data Handling

#### Statistical Model

We tested the hypothesized serial mediation model using Model 6 of the SPSS PROCESS macro. We selected PROCESS over structural equation modeling (SEM) for methodological precision. PROCESS directly tests theory-specified causal paths with observed variables [30], employs bias-corrected bootstrapping (5,000 samples) for reliable confidence intervals [31], and preserves statistical power by avoiding SEM’s latent construct complexity [32]. Linearity was confirmed via scatter plot inspection, revealing no meaningful nonlinearities.

#### Data Imputation for Sensitivity Analysis

To enable a robustness check comparing the clean data (n = 553) with the full sample (n = 737), we applied a structured imputation procedure to problematic cases:

Demographic Variables: We addressed missing values using mode imputation based on the distributions observed in the clean data subset [33].

Scale Items: We replaced missing values within each scale using the mean of the participant’s valid responses on that scale [34].

Logical Inconsistencies: We resolved implausible demographic values (e.g., age lower than organizational tenure) through logical inference (e.g., Age = 18 + Organizational Tenure) to retain internal coherence [35].

#### Measurement Model and Bias Assessment

We assessed the measurement model’s reliability and validity by calculating Composite Reliability (CR) and Average Variance Extracted (AVE). We then evaluated discriminant validity using Confirmatory Factor Analysis (CFA) in Mplus 7.0, comparing the theorized four-factor structure against alternative models. To examine common method bias (CMB), we performed Harman’s single-factor test via Principal Component Analysis in SPSS 25.0. We also applied procedural controls—such as temporal separation of predictors and outcomes and anonymity assurances—to reduce CMB at the design stage.

## Results

### Sample Characteristics

The final analytical sample consisted of 553 respondents. The sample was predominantly female (75.59%, n = 418). Participants’ ages ranged from 19 to 61 years (Mean = 41.76, Standard deviation = 8.62). Additional demographic information appears in Table 1.

**TABLE 1:** Sample description (Shandong, China. 2024).

Variable	Category	Frequency (n)	Percentage (%)
Age	19–30 years	87	15.73
	31–40 years	290	52.44
	41–50 years	111	20.07
	51–61 years	65	11.76
Marital status	Married	472	85.35
	Unmarried	81	14.65
Gender	Female	418	75.59
	Male	135	24.41
Education level	High school or below	5	0.91
	College diploma	51	9.22
	Bachelor’s degree	398	71.97
	Master’s degree or above	99	17.90
Occupation	Nurses	330	59.68
	Physicians	165	29.84
	Technicians	58	10.48
Organizational tenure	1–10 years	277	50.09
	11–20 years	173	31.28
	21–30 years	55	9.95
	31–41 years	48	8.68

N = 553.

### Statistical Power Assessment

Before testing hypotheses, we conducted a *post hoc* statistical power analysis using G*Power 3.1 for a linear multiple regression with four predictors [36]. With an alpha of 0.05, a sample size of 553, and the observed effect size (f^2^ = 0.59), derived from our model, the achieved statistical power was 0.95. This confirmed excellent power and effectively eliminated the risk of Type II error, ensuring the reliability of the subsequent analyses [36].

### Assessment of the Measurement Model

#### Reliability, Validity and Factor Analysis

All constructs demonstrated strong internal consistency, with CR values above 0.70 [37]. AVE values exceeded the 0.50 standard, confirming convergent validity [37]. Most factor loadings surpassed 0.70; two slightly lower items were retained due to theoretical relevance. The Kaiser-Meyer-Olkin (KMO) was 0.93, indicating excellent sampling adequacy. All Variance Inflation Factor scores were below 2.0, ruling out multicollinearity.

#### Common Method Bias Analysis

We assessed potential CMB using Harman’s single-factor test with unrotated PCA in SPSS 25.0. The first factor explained 47.87% of total variance, below the 50% threshold for serious CMB concerns [38]. This indicates that no dominant factor accounted for most covariance among variables. Procedural mitigation—such as guaranteed anonymity—further reduced CMB risk.

#### Confirmatory Factor Analysis

The CFA results strongly supported the distinctiveness of the four constructs. As shown in Table 2, the hypothesized four-factor model demonstrated excellent fit (*χ* ^*2*^/df = 3.04, CFI = 0.93, TLI = 0.92, RMSEA = 0.06, SRMR = 0.05) [39, 40], outperforming all alternative models and confirming discriminant validity.

**TABLE 2:** Confirmatory Factor Analysis result (Shandong, China. 2024).

Model	*χ* ^*2*^	*df*	*χ* ^*2*^ */df*	Comparative fit index	Tucker-lewis index	Root mean square error of approximation	Standardized root mean square residual
4 factors	550.80	181	3.04	0.93	0.92	0.06	0.05
3 factors Leader Tolerance + Collectivism, trust in colleagues, job satisfaction	934.09	184	5.08	0.87	0.85	0.09	1.00
3 factors Leader tolerance, collectivism, trust in Colleagues + Job satisfaction	1,504.48	186	8.09	0.77	0.74	0.11	1.00
2 factors Leader Tolerance + Collectivism, trust in Colleagues + Job satisfaction	1838.07	188	9.78	0.71	0.71	0.13	0.13
2 factors Leader Tolerance + Collectivism + Trust in Colleague,Job satisfaction	1726.43	188	9.18	0.73	0.73	0.12	0.09
1 factor	2,331.62	189	12.34	0.62	0.62	0.14	0.11

### Descriptive Statistics and Correlations

Table 3 reports the descriptive statistics and correlations. Leader tolerance correlated positively with collectivism (*r* = 0.34, *p* < 0.01), trust in colleagues (*r* = 0.58, *p* < 0.01), and job satisfaction (*r* = 0.48, *p* < 0.01). Collectivism also correlated positively with trust in colleagues (*r* = 0.52, *p* < 0.01) and job satisfaction (*r* = 0.43, *p* < 0.01). Trust in colleagues showed the strongest bivariate association with job satisfaction (*r* = 0.55, *p* < 0.01).

**TABLE 3:** Means, standard deviations and correlations of variables (Shandong, China. 2024).

Variable	Mean	Standard Deviation	1	2	3	4	5	6	7	8	9
1. Age	41.76	8.62	-	​	​	​	​	​	​	​	​
2.Gender^a^	1.76	0.43	−0.16 ^**^	-	​	​	​	​	​	​	​
3. Educational background^b^	3.06	0.56	0.03	−0.15 ^**^	-	​	​	​	​	​	​
4. Marital status^c^	1.87	0.34	0.24 ^**^	−0.03	0.15 ^**^	-	​	​	​	​	​
5.Organizational tenure	13.22	9.52	0.57 ^**^	0.00	−0.03	0.01	-	​	​	​	​
6. Leader Tolerance	3.43	0.86	0.00	0.07	0.01	−0.07	−0.03	(0.90)	​	​	​
7. Collectivism	4.07	0.74	0.13^**^	−0.04	0.01	0.04	0.09^*^	0.34^**^	(0.87)	​	​
8. Trust in colleagues	3.88	0.76	0.04	0.01	−0.04	−0.03	0.01	0.58^**^	0.52^**^	(0.95)	​
9. Job satisfaction	3.65	0.77	0.05	0.03	−0.02	−0.04	0.06	0.48^**^	0.43^**^	0.55^**^	(0.87)

N = 553.

^*^p < 0.05.

^**^p < 0.01.

^a^ For gender, 1 = male, 2 = female.

^b^ For educational background, 1 = high school and below, 2 = specialized secondary School, 3 = college, 4 = bachelor’s degree, 5 = master degree and above.

^c^ For marital status, 1 = single, 2 = married.

### Hypothesis Testing

We tested the chained mediation model using Hayes PROCESS Macro (Model 6) with 5,000 bootstrap samples to generate 95% confidence intervals (CIs) and ensure the robustness of findings. Tables 4, 5 summarize the results.

**TABLE 4:** Regression analysis (Shandong, China. 2024).

Variable	Model 1: Job satisfaction			Model 2: Collectivism (direct Effect)			Model 3: Trust in colleagues (direct Effect)			Model 4: Job satisfaction (full Model)		
	β	b	t-value	β	b	t-value	β	b	t-value	β	b	t-value
Predictors												
Age	0.02	0.00	0.35	0.07	0.01	1.45	0.00	0.00	0.03	−0.01	−0.00	−0.13
Gender^a^	−0.01	−0.01	−0.13	−0.05	−0.09	−1.26	−0.01	−0.02	−0.42	0.01	0.03	0.42
Educational Background^b^	−0.03	−0.04	−0.72	−0.01	−0.01	−0.25	−0.05	−0.07	−1.51	−0.01	−0.01	−0.26
Marital Status^c^	−0.01	−0.02	−0.27	0.04	0.09	1.02	−0.01	−0.01	−0.18	−0.02	−0.05	−0.60
Organizational Tenure	0.06	0.01	1.32	0.05	0.00	1.09	−0.01	−0.00	−0.21	0.05	0.00	1.13
Leader tolerance	0.48	0.43	12.69^***^	0.35	0.30	8.78^***^	0.46	0.40	13.48^***^	0.23	0.21	5.40^***^
Collectivism	​	​	​	​	​	​	0.37	0.38	10.81^***^	0.18	0.19	4.52^***^
Trust in colleagues	​	​	​	​	​	​	​	​	​	0.32	0.32	6.81^***^
Model summary												
R	0.48			0.37			0.68			0.61		
R^2^	0.23			0.14			0.46			0.37		
F-value	27.77			14.60			66.14			39.08		

N = 553.

***p < 0.001.

^a^ For gender,1 = male, 2 = female.

^b^ For educational background, 1 = high school and below, 2 = specialized secondary School, 3 = college, 4 = bachelor’s degree, 5 = master degree and above.

^c^ For marital status, 1 = single, 2 = married.

**TABLE 5:** Mediation effect analysis (Shandong, China. 2024).

Path	Effect	Standard error	95% confidence interval	
			Lower limit of confidence interval	Upper limit of confidence interval
Total effect	0.43	0.03	0.36	0.50
Direct effect	0.21	0.04	0.13	0.28
Indirect effect	0.22	0.03	0.17	0.29
Indirect path 1: Leader tolerance → collectivism → job satisfaction	0.06	0.02	0.03	0.09
Indirect path 2: Leader tolerance → trust in colleagues → job satisfaction	0.13	0.03	0.08	0.19
Indirect path 3: Leader tolerance → collectivism → trust in colleagues → job satisfaction	0.03	0.01	0.02	0.06

N = 553. Bootstrap n = 5,000. In this table, the Effect is reported in terms of unstandardized coefficients (*b*) only, to reflect the actual magnitude of the effects. For standardized coefficients (*β*), please refer to the text description.

We first assessed the direct effect of leader tolerance on job satisfaction (Table 4). As expected, leader tolerance demonstrated a strong and significant total effect on employees’ job satisfaction (*β* = 0.48, *b = 0.43, p < 0.001*). After introducing the mediators, the direct effect remained statistically significant (*β* = 0.23, *b = 0.21, p < 0.001*), fully supporting Hypothesis 1.

Next, we examined collectivism as a mediator. Leader tolerance significantly predicted employees’ collectivism (*β* = 0.35, *b = 0.30, p < 0.001*), and collectivism in turn significantly predicted job satisfaction (*β = 0.18, b = 0.19, p < 0.001*) (Table 4). The indirect effect of leader tolerance on job satisfaction via collectivism was significant (*β = 0.06, b = 0.06*, 95% CI [0.03, 0.09]), providing strong support for Hypothesis 2.

We then tested the mediating role of trust in colleagues. Leader tolerance strongly predicted trust (*β = 0.46, b = 0.40, p < 0.001*), and trust significantly predicted job satisfaction (*β = 0.32, b = 0.32, p < 0.001*) (Table 4). The indirect effect via trust in collagues was substantial (*β = 0.14, b = 0.13*, 95% CI [0.08, 0.18], Table 5), fully supporting Hypothesis 3.

The chained mediation path analysis further revealed that collectivism significantly predicted trust in colleagues (*β = 0.37, b = 0.38*, *p < 0.001*), yielding an indirect effect of *β* = 0.04, *b* = 0.03, and a 95% CI of [0.02, 0.06] (Table 5).

**FIGURE 2:**
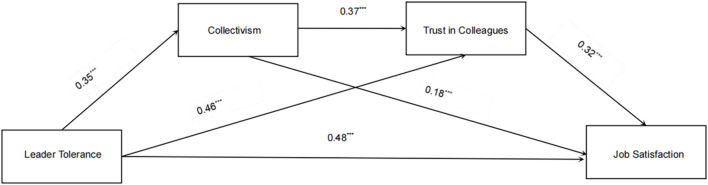
Sequential mediating effects of collectivism and trust in colleagues on leader tolerance and job satisfaction (Shandong, China. 2024).

Figure 2 illustrates the full sequential mediation pathway. The combined indirect effects across H2, H3, and H4 were significant (*β = 0.25, b = 0.22*, 95% BootCI [0.17, 0.29]), accounting for 51.16% of the total effect of leader tolerance. Trust in colleagues emerged as the strongest mediator, contributing 30.23% of the total effect. Thus, all Hypotheses (H1–H4) received full and robust support.

### Sensitivity Analysis Results

We conducted a comprehensive sensitivity analysis to test the robustness of the findings against potential data quality issues, comparing results from the clean dataset (n = 553) with the full sample (n = 737), which included logically corrected and imputed responses. This analysis ensured that the estimated relationships remained theoretically valid and statistically stable across different data treatment strategies. The imputation protocol, detailed in Section *Data Imputation for Sensitivity Analysis*, was applied in a structured and conceptually grounded manner.

The confirmatory factor analysis confirmed strong and comparable measurement validity across both samples. The clean data produced *χ* ^*2*^/df = 3.04, CFI = 0.93, TLI = 0.92, RMSEA = 0.06, SRMR = 0.05, while the full sample produced *χ* ^*2*^/df = 3.64, CFI = 0.94, TLI = 0.93, RMSEA = 0.06, SRMR = 0.04. All fit indices remained within acceptable thresholds. Harman’s single-factor test showed 47.87% variance explained in the clean data and 56.98% in the full sample—both under the 60% threshold [41], indicating that common method bias posed no meaningful threat.

The chain mediation model (leader tolerance → collectivism → trust in colleagues → job satisfaction) remained highly consistent across data treatments. In the clean data, the total indirect effect was 0.23 (95% CI: 0.17–0.29); in the full sample, it was 0.28 (95% CI: 0.23–0.34). Equally, the chained mediation effect also remained stable and statistically significant—0.04 (95% CI: 0.02–0.06) in the clean data and 0.05 (95% CI: 0.03–0.07) in the full sample.

Controlling for demographics did not alter the interpretive pattern. The total indirect effect was 0.23 (95% CI: 0.17–0.29) in the clean sample and 0.29 (95% CI: 0.23–0.35) in the full sample. Besides, the completely standardized chain mediation effect remained consistent at 0.04 (clean) versus 0.06 (full), even without controls.

Overall, the near-identical patterns across data scenarios provide compelling evidence of empirical stability and theoretical robustness. The clean-data-first imputation strategy successfully preserved data validity over sample inflation, and the persistence of leader tolerance’s indirect effects across all models confirms that the identified mechanisms reflect genuine psychological processes rather than artifacts of data treatment.

## Discussion

This study clarifies the psychological mechanism linking leader tolerance to job satisfaction among healthcare professionals. By validating a sequential social-psychological pathway, it advances understanding of how leadership activates resource dynamics within public health teams.

### Key Findings

This study yields four key findings that address existing research gaps. First, leader tolerance directly enhances job satisfaction (*β* = 0.23, *p* < 0.001) while also operating through indirect pathways, confirming its role as a multidimensional resource. Second, trust in colleagues emerged as the strongest mediator (indirect *β* = 0.14), highlighting the importance of relational resources. Third, the sequential mediation pathway was validated (*β* = 0.04), showing leader tolerance fosters collectivism, which in turn builds trust. Fourth, over half of the total effect (51.16%) was explained by these indirect pathways, demonstrating leadership operates primarily through multi-stage resource activation. Sensitivity analysis confirmed the robustness of these findings across data treatments.

### Theoretical Contributions

This study makes four major theoretical contributions. First, it advances public health leadership theory by empirically establishing a staged resource propagation pathway—from leader tolerance to collectivism to trust to job satisfaction—moving beyond simple direct-effect models. While previous research has demonstrated connections between supportive leadership and employee wellbeing [42], these findings extend this work by illuminating the specific sequential mechanism through which these effects manifest. In addition, it addresses a critical gap in understanding how leadership practices translate into improved psychological outcomes through social resources.

Second, it extends COR theory by showing how leader tolerance initiates a resource gain spiral, demonstrating how leadership triggers collective resource caravan formation, not just individual-level resource accrual. Our finding, which reveals that leader tolerance initiates a resource gain spiral, provides deeper insight into resource caravan formation in healthcare contexts. This extends COR theory’s application beyond individual resource dynamics to demonstrate how leadership creates collective resource reservoirs.

Third, it bridges macro-level leadership behavior and micro-level team interactions. It shows that leader tolerance translates into interpersonal resource exchange—for example, turning an error into a learning-centered team dialogue rather than a blame-oriented response. While previous studies have predominantly focused on individual-level outcomes, this model links leadership practices with interpersonal resource exchanges. For instance, when a leader responds to a work error by facilitating a blameless team review. Thus, this demonstration of tolerance strengthens both collective identity and interpersonal trust.

Fourth, it identifies leader tolerance as a critical leadership competency in high-stakes domains such as infectious disease outbreaks, aging populations, health equity, climate emergencies, and digital health system transformation. In these contexts, leader tolerance promotes psychological safety and enhances the collective efficacy of public health teams, facilitating coordination and responsiveness to community needs. Taken together, these contributions collectively establish a theoretical framework applicable across various public health settings, promoting effective leadership practices and workforce stability.

While robust, this study acknowledges the limitation that quantitative analysis cannot fully capture how tolerance manifests in real-time practice. Future mixed-methods research could reveal context-specific expressions of tolerant leadership across varied public health systems.

### Practical Implications

This study proposes a four-phase implementation framework to help public health organizations enhance job satisfaction and improve community health outcomes through a supportive work environment.

#### Phase 1: Establish Psychological Safety Via Leader Tolerance

Organizations should develop leadership programs that reframe errors as learning opportunities. Training leaders to conduct blameless debriefings—focusing on “what can we learn?” rather than “whose fault was this?”—fosters the psychological safety necessary for team resilience.

#### Phase 2: Build Collective Identity Before Trust

Team-building should first strengthen shared identity. Implementing inter-departmental projects with superordinate goals and peer recognition systems for collective achievements makes a “we-first” group identity salient, creating the essential foundation for trust.

#### Phase 3: Convert Collectivism Into Relational Trust

With a collective identity established, targeted practices like cross-role simulations and peer mentorship can be effectively introduced. These initiatives build empathy, demonstrate competence, and deepen interpersonal confidence among team members.

#### Phase 4: Institutionalize A Learning Culture

Organizations must transition from a punitive to a learning-oriented culture. This involves implementing user-friendly error-reporting systems and dedicating time for team reflection. The cultural shift should be monitored through pulse surveys tracking perceptions of leader tolerance, collectivism, and trust, enabling proactive interventions.

This sequential, evidence-based blueprint provides a concrete pathway to simultaneously strengthen workforce resilience and patient-care quality, moving beyond generic advice to offer an actionable strategy for public health challenges.

### Limitations and Future Directions

This study examines the effects of leader tolerance in healthcare while acknowledging several limitations. The cross-sectional design limits causal inference, and the reliance on self-reported data introduces potential common method bias. Additionally, the single-country institutional context may restrict generalizability across diverse healthcare systems or cultural norms. Future research should employ longitudinal or experimental designs, incorporate objective performance indicators, and test cultural or individual-level moderators such as professional autonomy or cultural tightness–looseness. A mixed-methods approach—combining surveys with interviews, ethnographic field observations, or real-time error-tracing—would provide richer contextual insights into how leader tolerance is enacted during critical incidents.

Based on our findings, we, therefore, recommend that public health organizations actively implement leadership development programs that explicitly cultivate error-tolerant behaviors, as such leadership demonstrably strengthens collectivism, reinforces interpersonal trust, and enhances job satisfaction in high-pressure clinical environments. Future studies should also explore how tolerant leadership interacts with digital health systems, AI-supported diagnosis, or interprofessional collaboration platforms to further amplify resilience, reduce fear-based reporting, and accelerate team learning dynamics.

### Conclusion

This study demonstrates that leader tolerance for errors can catalyze a transformational shift within clinical teams, positioning them as resilient learning communities rather than compliance-driven task units. By intentionally fostering collectivism and trust in colleagues, tolerant leadership reframes errors as shared learning opportunities instead of personal failures. This psychological reframing enhances job satisfaction and supports the delivery of higher-quality patient care. Our findings, therefore, offer a practical and theoretically grounded road-map for designing psychologically safe, learning-oriented healthcare environments driven by empathetic, future-focused leadership. Such leadership is not only essential for daily operational stability but also foundational for long-term public health resilience in the face of crises, innovation demands, and evolving global health challenges.

## Data Availability

The data that support the findings of this study are available upon request from the corresponding author.
